# Supplementary material for: Iron activates microglia and directly stimulates indoleamine-2,3-dioxygenase activity in the N171-82Q mouse model of Huntington’s disease
Source: PLoS One. 2021 May 14;16(5):e0250606. doi: 10.1371/journal.pone.0250606 (PMC8121302; doi:10.1371/journal.pone.0250606)
Supplement: Attachment — Submitted filename: ResponseToReviewers.pdf [file pone.0250606.s001.pdf]

Dear editor and reviewers: We appreciate the input on our manuscript. Responding to this has enabled us to make further improvements. Please see the specific comments below that are responses to all the reviews comments.

We hope that you will now find this manuscript acceptable for publication in PLOS ONE.

Sincerely, Jonathan Fox

Line numbers refer to the finalized version without track changes.

Reviewer 1:

Major points:

1. The reviewer raises the question of whether loss of brain weight and striatal volume is sufficient to support the conclusion that there is neurodegeneration, or would brain/striatal atrophy be a more appropriate term to use. This a valid point. We do think there is significant precedence in the literature that our findings are sufficient to use the term "neurodegeneration". There is overwhelming evidence in human HD that brain atrophy is driven to a large extent by neuron degeneration and loss (Curr Top Behav Neurosci. 2015;22:33-80). This may be also contributed by oligodendrocyte degeneration in white matter (PNAS May 7, 2019 116 (19) 9622-9627). The N171-82Q mouse model of HD demonstrate a number of changes that support neuron degeneration including TUNEL and silver stain positive neurons in striatum and or cortex (Curr Top Behav Neurosci. 2015;22:33-80; Neurobiology of Disease, June, 2008, 30;293-302). This is in addition to very well established loss of striatal and overall brain mass (Brain Pathol 2016 Nov;26(6):726-740). While we acknowledge that demonstrating neuron-specific changes would have enhanced the study, our interpretation of neurodegeneration is consistent with the current literature. To address this point in the text we have made the following addition. "These mice develop neurodegeneration as evidenced by loss of brain and striatal volume, and neuronal loss" and included a reference. Line 91.
2. Figs 1E and 1F have now been modified according to the reviewer's suggestions.
3. EOC 20 has now been changed to "an immortalized microglial cell line". See lines 135 and 395.
4. Statement about CCL activating microglia – We agree with the reviewer that the original statement was inaccurate. We have now changed this to -"The chemokine CCL2 (MCP-1), expressed by both neurons and glia, recruits microglia and peripheral monocytes to sites of inflammation in the central nervous system. Expression of CCL2 is associated with microglial activation, but is an indicator of a more generalized, sustained inflammatory response". See lines 207-210.

Minor points:

1. KP is now defined correctly in the abstract.
2. We have now explained what deferoxamine does. We changed the sentence to "IDO activity in HD mice that received NIS was significantly decreased by the potent iron (III) chelator deferoxamine added *ex vivo* in striata ($p = 0.0067$) and cortices ($p = 0.0028$)". See lines 100-101.
3. We have now made the terminology around iron more consistent. We have removed the term ferrous and used "iron (II)" instead. Most of the time we do not designate the oxidation state of iron because it is not necessary, or the form of iron is unknown.
